# 

**Published:** 1887-04-09

**Authors:** 


### THE HOSPITAL

An Institution, Family, and Parochial Journal of Hospitals, Asylums, & all Agencies for the Care of the Sick, Criticism & News.

### CONTENTS.

**Sleep and Sleeplessness** - - - - 17
**Words of Conso'ation.**—XXVIII. Death - - 18
**Nurse Marion's Romance.** By Kate Furley. Chapter III. Jack Holtayne in Hospital - - - 19
**Moral and Physical Massage.** By Dr. Stretch Dowse. I. Explanatory and Descriptive - - 21
**Small Economies in Cottage Hospital Management.** By a Cottage Hospital Matron, and Miss Mattocks - - - - - 23
**Notes and News.**— Miscellaneous Items. — Hospital Enlargement.— Lecture in Aid of the Royal Hospital for Children. — New Hospital for Glasgow. — Annual Report of the Shrophire and Wales Eye Hospital — Vacancies.—"Give your Gold to the Hospitals."—The North-West London Hospital.—The Wolverhampton and Staffordshire General Hospital.—The Bristol Royal Infirmary. — Donations, Legacies, etc., to Medical Charities.—The Midwives' Institute and Trained Nurses' Club.—Convalescent Home for Working Men.—The Children's Hospital at Chelsea.—Lunacy in Russia, etc. 24
**Annotations.**—The National Pension Fund.—Private Wards for Lying-in Cases.— Hospital Locum Tenens - 27
**Chats about Medical Museums.** By One of the Crowd.—No. III - - - - - - 28
**Sydney Smith as a Doctor** - - - - - 29
**Public and Private Nursing Institutions.** By Mrs. Bedingfeld, and a Provincial Hospital Matron - - - - - - - 30
**The Advertisements of Endowed Hospitals** 31
**In- and Out-Patients' Hospital Diary** - - 32
**The Children's Corner.**—Puzzles, Answers, etc. - vi
**Amusements with Profit** - - - - - vi

